# A comparative analysis of suture-augmented and standard hamstring autograft single-bundle ACL reconstruction outcomes: short-term functional benefits without long-term impact

**DOI:** 10.1186/s12891-023-07100-7

**Published:** 2023-12-15

**Authors:** Reza Tavakoli Darestani, Sina Afzal, Ali Pourmojarab, Mojtaba Baroutkoub, Shahram Sayyadi, Hasan Barati

**Affiliations:** 1 Department of Orthopedic Surgery, School of Medicine, Shahid Beheshti University of Medical Sciences, Tehran, Iran; 2 Anesthesiology Research Center, Shahid Beheshti University of Medical Sciences, Tehran, Iran

**Keywords:** Anterior cruciate ligament, Reconstruction, Augmentation, Patient reported outcome measures

## Abstract

**Background:**

Augmentation of the biologic graft with nonabsorbable suture material during anterior cruciate ligament reconstruction (ACLR) is a relatively new technique to enhance its biomechanical properties and add additional support to the critical process of healing. We aimed to compare the short-term functional patient-reported outcome measures (PROMs) and complication rates of patients treated with either standard single-bundle four-strand hamstring ACLR or added suture augmentation (SA).

**Methods:**

Patients undergoing arthroscopic ACLR between February 2015-January 2017 and in the standard ACLR group, and between February 2017-September 2019 in the SA-ACLR group operated by adding a no.5 FiberWire® (Arthrex, Naples, FL, USA) braided suture to the hamstring autograft, were retrospectively reviewed and the PROMs were compared. Patients were followed up for a 24-month period and PROMs were assessed by the International Knee Documentation Committee (IKDC) Subjective Knee Form and Tegner-Lysholm knee score. Patients’ demographic and clinical characteristics, and postoperative complications including graft retear requiring revision surgery, deep vein thrombois, and surgical site infection were recorded and analyzed.

**Results:**

We included 79 patients with mean age of 31.6 ± 8.3 years in the standard ACLR group, and 90 patients with mean oge of 30.5 ± 7.6 in the SA-ACLR group. There was no statistically significant difference between the two groups in terms of age, sex, body mass index, and medical comorbidities. The values of the IKDC scores increased to 75.8 ± 18.9 in the standard ACLR group, and 85.6 ± 12.6 in the SA-ACLR group, 24 months after the operation (*P* < 0.05). The 24-month postoperative Tegner-Lysholm scores escalated to 79.3 ± 21.0 in the standard ACLR group and 91.0 ± 13.7 in the SA-ACLR group (*P* < 0.05). Four (5.1%) patients in the standard ACLR group and 4 (4.4%) in the SA-ACLR group experienced graft retear requiring revision surgery (*P* > 0.05). Incidence of surgical site infection and deep vein thrombosis showed no significant differences between the two groups, 24 months after ACLR.

**Conclusion:**

SA-ACLR is associated with improved short-term functional PROMs compared to the standard hamstring ACLR. Although SA did not reduce the retear rate, and infection and DVT rates did not differ between study groups, superior improvement of PROMs in SA approach, leverages this method for ACLR.

## Background

As the main restraint against the anterior translation of the tibia, the anterior cruciate ligament (ACL) portrays a crucial role in human knee kinematics and stability [1, 2]. ACL injury with an incidence rate of 1 in 50 male and 1 in 29 female athletes [3], is one of the most prevailing and debilitating knee injuries in young athletes [4, 5]. Reconstruction of the ruptured ACL and repairing the associated injuries to the menisci, may revive native knee kinematics and lead to enhanced short- and long-term patient-reported outcomes [6, 7].

The most common autografts used for ACL reconstruction (ACLR) are bone-patellar tendon-bone, hamstring tendon, and quadriceps tendon grafts, which all of them have almost similar reconstruction outcomes based on literature [8]. Augmentation of the graft in ACLR either by biological or synthetic materials has shown to be successful in improving the biomechanical stability of the ACL graft and reducing adverse outcomes of ACLR like ligament retear [9, 10]; however, detabes still exist on the efficacy of this method [11–13]. The main purpose of graft augmentation is to support the tissue during the critical phase of remodeling and revascularization, and potentially decrease the risk of graft retear [14, 15].

Patient-reported outcome measures (PROMs) is a rising cornerstone of surgical care in orthopedics with several beneficiaries for clinical decision-making and patient-centered care [16]. Some studies have demonstrated enhanced PROMs and earlier recovery in terms of level of activity and knee stability following augmentated ACLR [8, 9]. A recent scoping review of studies reported superior biomechanical and functional outcomes of augmented ACLR, and notably no increase in postoperative complications following this approach [10].

As a relatively new concept, most pulications have focused on the surgical technique, while few studies focused on the clinical outcomes of the augmented ACLRs [9]. Also, the evidence on the comparison of results of suture augmentation (SA) ACLR with standard methods of ACLR without any augmentation, is scarce [15]. In this regard, the present study aimed to investigate the short-term functional outcomes and complications of SA versus standard hamstring ACLR to reveal the clinical outcomes of this surgical amendment for its further application in future.

## Materials and methods

### Study design and population

By a retrospective design, the data of all arthroscopically-assisted ACLRs performed and coded as ‘‘ACL surgery’’ between February 2015 and September 2019 in a tertiary orthopedic surgery center in Iran were reviewed. All patients signed a written informed consent prior to their surgery. All procedures were performed in accordance with the Declaration of Helsinki and were approved by the research ethics committee of School of Medicine at Shahid Beheshti University of Medical Sciences, Tehran, Iran (code: IR.SBMU.MSP.REC.1400.379). The exclusion criteria were age younger than 18 years, ACL primary repairs, revision surgeries, two-stage surgeries, a contralateral ACL tear or knee laxity, concomitant contra- or ipsilateral injury to the posterior cruciate ligament, collateral ligaments, or posterolateral complex, acute fracture, limb malalignment requiring simultaneous corrective osteotomies, follow-up of less than 24 months, and use of any types of graft other than hamstring autograft. All surgeries were performed solely by one board-certified senior orthopedic surgeon. Surgeries before February 2017 were all performed by the standard hamstring autograft four-strand single-bundle ACLR approach. Since February 2017, the surgeon started adding the SA technique to the standard ACLR method. By this approach, two comparative cohorts of patients undergoing standard ACLR using hamstring grafts (February 2015-January 2017) or SA-ACLR (February 2017-September 2019) were included as the comparison groups in this study.

### Surgical technique

**Fig. 1:**
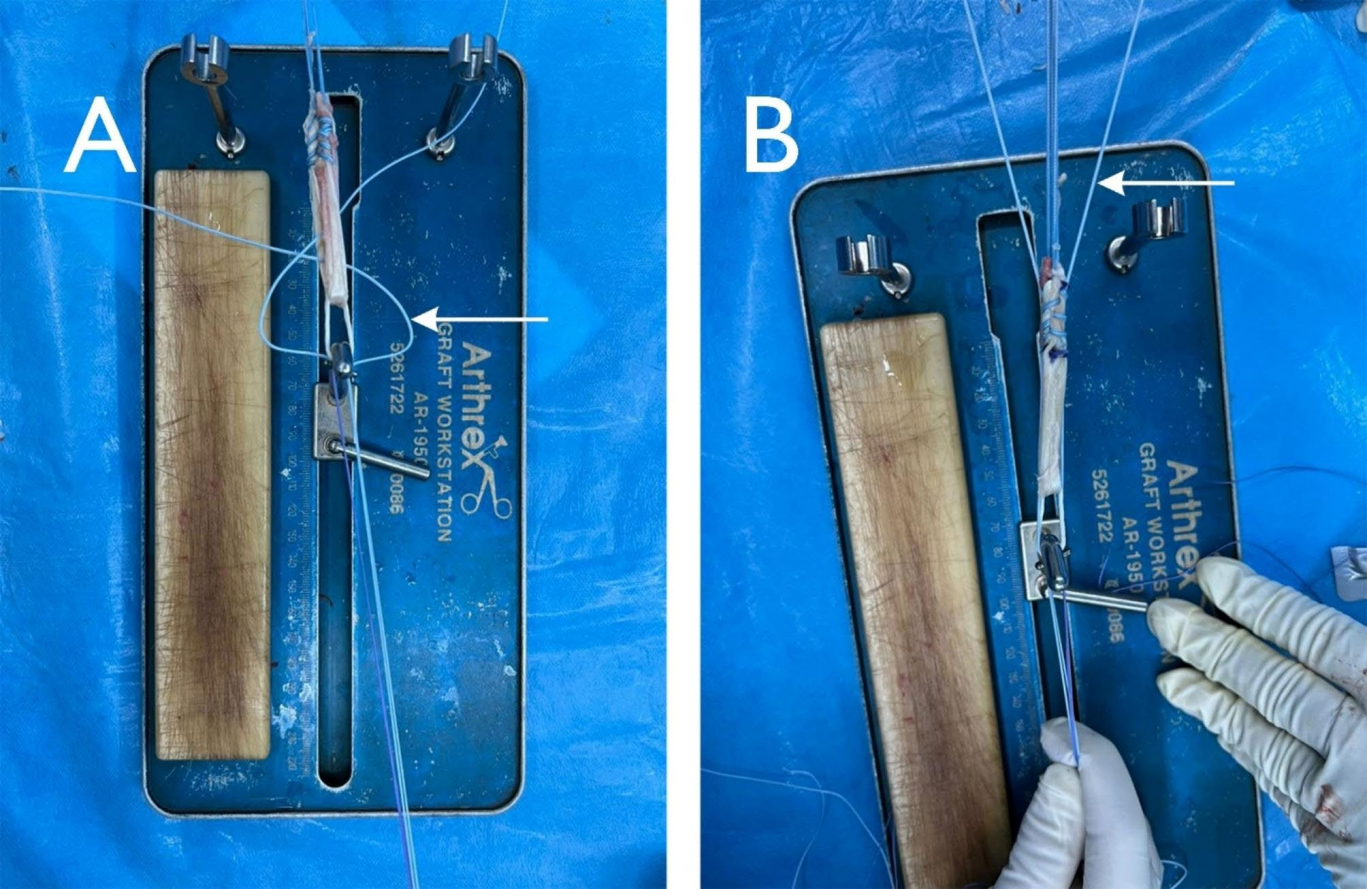
A no.5 Fiberwire suture (white arrow) was passed through the titanium button of the fixation device and was crossed once at the femoral loop end (**A**), and for the second time at the tibial end (**B**)

With the patient in the supine position and under spinal or general anesthesia, first we did a comprehensive physical examination of the injured knee including the pivot-shift, anterior drawer, and Lachman tests. Then the prophylactic intravenous antibiotics were injected, a high-thigh sterile pneumatic tourniquet was applied, and the limb was draped in a sterile fashion. Initially, diagnostic knee arthroscopy was performed through standard anterolateral and anteromedial portals, and any meniscal or chondral pathologies were approached accordingly. Upon confirmation of the complete tear of ACL, a 4–5 cm long oblique incision on the anteromedial aspect of the proximal tibia was made followed by the identification of the pes anserinus. In all of our cases, only the semitendinosus tendon was harvested and quadrupled to produce the graft with a minimum of 7 mm thickness and 6.5 cm length. None of the cases required further harvesting of the gracilis to achieve the desired graft length or diameter. The tendon was then transferred to a graft preparation station (Arthrex, Naples, FL, USA) and tensioned. Two ends of a no. 5 FiberWire® (Arthrex, Naples, FL, USA) braided ultra-high molecular weight polyethylene (UHMWPE) suture was passed through the titanium femoral suspensory fixation device (Retrobutton®, Arthrex, Naples, FL, USA) running alongside the graft and was crossed two times. Once at the femoral loop-end of the graft and then at the tibial end, the graft strands were stitched together using heavy absorbable sutures (Fig. 1).

**Fig. 2:**
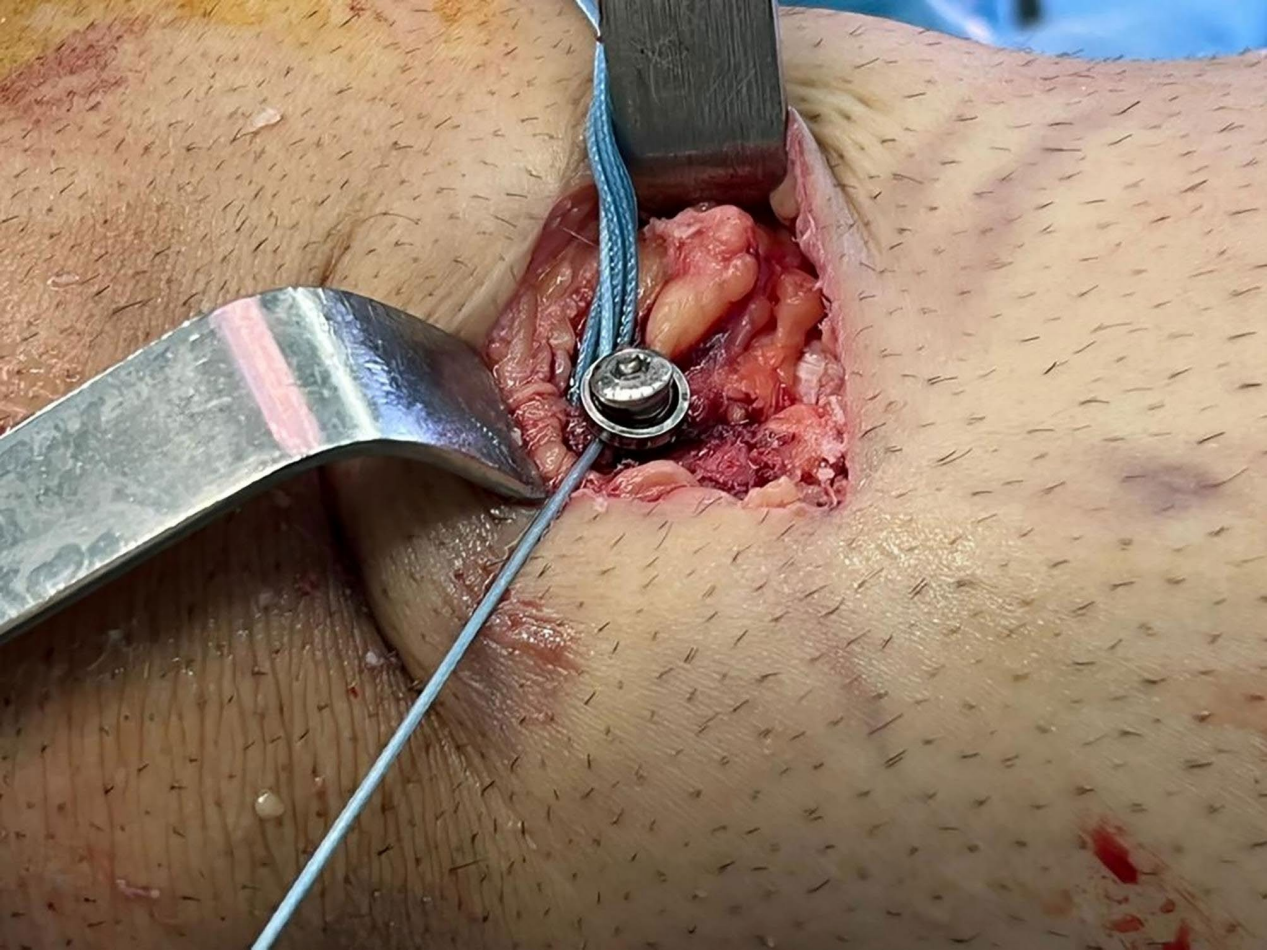
The augmentation suture was secured to the anteromedial of proximal tibia using a 3.5 mm cortical screw and washer

**Fig. 3:**
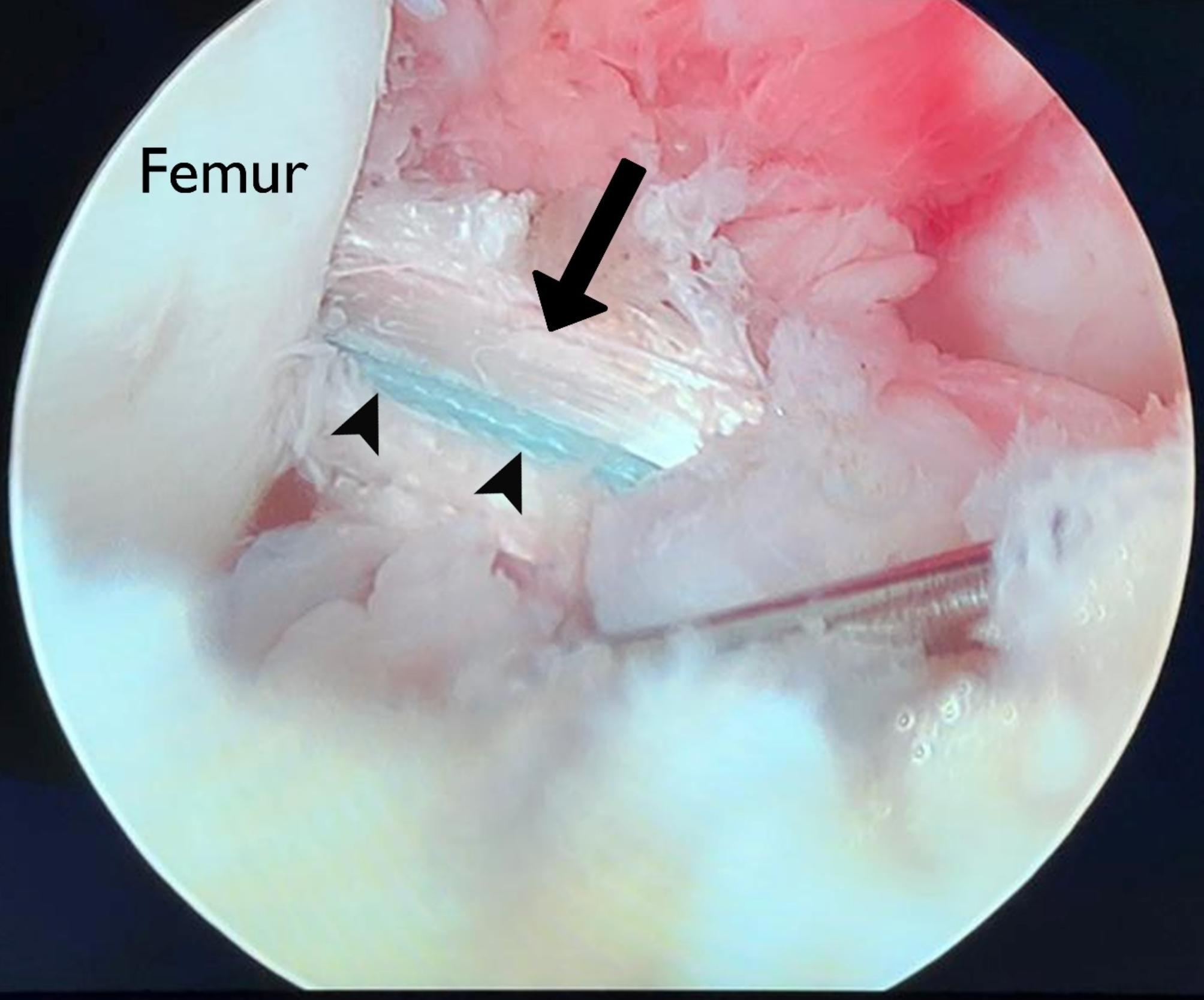
Final arthroscopic view showing the hamstring graft (black arrow) and the synthetic suture augment (black arrowheads) in place

Next, the femoral and tibial tunnels were reamed and the femoral fixation device was passed and fliped on the cortical bone under arthroscopic control. Then, the suture augment was fixed using a 3.5 mm stainless steel fully threaded cortical screw and washer to the anteromedial proximal of tibia, 2–3 cm below the tibial tunnel in full extension of the knee, under manual tension (Fig. 2). The tibial side of the graft was then secured with a biocomposite interference screw (Arthrex, Naples, FL, USA) in 30 degrees flexion of the knee with a posterior drawer force under proper tension of the graft, making sure that the tension of the suture was not higher than that of the graft. By independently controlling the tension in the biological component (the graft) and the synthetic component (the fibertape suture), we aimed to minimize the risk of excessive tension on the graft while avoiding stress shielding of the graft. Finally, a re-arthroscopy was done to ensure the proper tension of the graft-suture composite through the full knee range of motion (ROM) (Fig. 3). The surgical incision was closed in a standard manner after completing of all abovementioned steps.

### Post-operation care and patient follow-up

Patients were discharged the following day after surgery, and active and passive knee ROM and ankle pumps were initiated and encouraged on the discharge day. The patients received no oral antibiotics and all were prescribed 81 mg of daily acetylsalicylic acid (ASA) for 28 days as prophylaxis for deep vein thrombosis (DVT). The initial postoperative period was uneventful for all of the patients. A standard evidence-based rehabilitation protocol by a physical therapist was started for patients since postsurgical week 1, as described by Grinsven et al. [17]. Patients were followed up for 1 month, 3 months, 6 months, one year after surgery, and every 6 months thereafter for a minimum of 24 months.

### Study variables and data collection

PROMs were the primary outcomes assessed in this study. Twenty-four months after the operation, the International Knee Documentation Committee (IKDC) Subjective Knee Form and Tegner-Lysholm knee score were used for assessment of PROMs and the results were compared to the preoperative status of each patient. The IKDC score is an internationally validated patient-oriented questionnaire with two subscales of knee symptoms and knee articulation, and activity level, used to evaluate patients with ligamentous and meniscal injuries of knee [18]. The validity and reliability of the IKDC tool for Iranian patients with ACL and meniscal surgeries has been examined before [19, 20]. The Lysholm knee score is mainly used to assess patients with ligamentous injuries of knee [21], and the Tegner score was developed to compensate for the weaknesses of the Lysholm scale [22]. The Tegner-Lysholm scale is composed of eight different items including subscales for pain, instability, locking, swelling, limp, stair climbing, squatting, and the need for support, all scored in four grades of excellent, good, fair, and poor by patient [23]. All questionnaires were completed by one trained research assistant who was blind to the method of surgical treatment. Age, sex, body mass index (BMI), graft type, concomitant meniscal injury, surgery on the dominant limb, smoking, and comorbidities were the demographic and clinical variables inculeded in analyses. Complications after surgery including documented graft retear requiring revision surgery, DVT proved by Doppler ultrasound after clinical suspicion, and surgical site infection requiring intervention were recorded.

### Statistical analysis

Categorical variables were summarized in frequency and percentage, and continuous variables were described using mean and standard deviation (± SD). To compare the means of the quantitative factors before and after surgery, the paired t-test was used. Analysis of variance (ANOVA) was used to investigate differences in pertinent patient characteristics between the two surgical groups. A 2-tailed P-value of less than 0.05 was considered as the statistical significance level. The statistical analysis was performed using SPSS software Version 25.0 (IBM SPSS Statistics for Windows, Version 16.0, IBM Corp.).

## Results

Initially, data from 284 patients were extracted. Finally, a total of 169 patients met the eligibility criteria and entered the study in the two groups of standard ACLR (79 patients) and SA-ACLR (90 patients). In the standard ACLR group, 72 (91.1%) patients were male and 7 (8.9%) were female. In the SA-ACLR group, 88 (97.8%) patients were male and 2 (2.2%) were female. The mean age of patients included in the standard ACLR and SA-ACLR groups were 31.6 ± 8.3 and 30.5 ± 7.6 years, respectively. The mean BMI of patients was 27.5 ± 4.9 kg/m^2^ in the standard ACLR group, and 26.3 ± 4.0 in the SA-ACLR group. Sixteen (20.3%) patients in the standard ACLR group and 17 (18.9%) in the SA-ACLR group were everyday smokers. Six (7.6%) patients in the standard ACLR group and 4 (4.4%) in the SA-ACLR group had a known comorbidity; including, diabetes mellitus, hypertension, hyperthyroidism, asthma, IgA nephropathy, and psoriasis.

In the standard ACLR group, 42 (53.2%) surgeries were performed on the right knee and 37 (46.8%) on the left knee, whereas in the SA-ACLR group these numbers were 48 (53.3%) and 42 (46.7%) for the right and left knees, respectively. Forty-two (53.2%) patients in the standard ACLR group and 38 (42.2%) in the SA-ACLR group had a concomitant medial or lateral meniscal injury that was approached during the surgery, either by primary repair or partial meniscectomy. The variations of the demographic and clinical characteristics were not statistically significant between the two study groups as summarized in Table 1.

**Table 1:** Demographic and clinical characteristics of patients in the two study groups

Variable^*^	Standard ACLR Group (N = 79)	SA-ACLR Group (N = 90)	*P*-value
Age (years)	31.6 (8.3)	30.5 (7.6)	0.355
Sex	Male: 72 (91.1%) Female: 7 (8.9%)	Male: 88 (97.8%) Female: 2 (2.2%)	0.055
BMI (kg/m^2^)	27.5 (4.9)	26.3 (4.0)	0.068
Meniscal Injury	42 (53.2%)	38 (42.2%)	0.155
Smoking	16 (20.3%)	17 (18.9%)	0.823
Patients with comorbidities	6 (7.6%)	4 (4.4%)	0.386
Operated knee side	Right: 42 (53.2%) Left: 37 (46.8%)	Right: 48 (53.3%) Left: 42 (46.7%)	0.982

^*^Constant variables are reported as mean (standard deviation) and categorical variables are reported in number (percent). BMI: body mass index, ACLR: anterior cruciate ligament reconstruction, SA-ACLR: suture augmentation anterior cruciate ligament reconstruction

Regarding the PROMs, the mean preoperative IKDC scores for the standard ACLR and SA-ACLR groups were 30.2 ± 7.2 and 31.1 ± 6.6, respectively. Twenty-four months after surgery, the values of the IKDC scores increased to 75.8 ± 18.9 in the standard ACLR group, and 85.6 ± 12.6 in the SA-ACLR group, which was statistically significant (*P* < 0.05) compared to the preoperative status of each group. While the between groups preoperative IKDC score did not differ significantly (*P* = 0.429), the postoperative IKDC score of the SA-ACLR group was significantly higher than the standard ACLR group (*P* < 0.05). The mean preoperative Tegner-Lysholm scores were 43.5 ± 8.2 and 42.3 ± 10.3 in the standard ACLR and SA-ACLR groups, respectively. The 24-month postoperative scores escalated to 79.3 ± 21.0 in the standard ACLR group and 91.0 ± 13.7 in the SA-ACLR group. Similar to the IKDC score, the within group analysis pre- and post-operatively showed statistically significant changes (*P* < 0.05). While the preoperative between-group Tegner-Lysholm scores were not significantly different (*P* = 0.392), the observed difference in the postoperative scores of this measure was statistically significant (*P* < 0.05) (Table 2).

**Table 2:** Patient-reported outcome measures at 24 months after the reconstructions

Variable^*^	Time point	Standard ACLR Group (N = 79)	SA-ACLR Group (N = 90)	*P*-value
IKDC score	Preoperative	30.23 (7.24)	31.08 (6.64)	0.429
	Postoperative	75.75 (18.91)	85.62 (12.60)	< 0.05
Tegner-Lysholm score	Preoperative	43.53 (8.21)	42.29 (10.3)	0.392
	Postoperative	79.30 (21.00)	90.98 (13.70)	< 0.05

^*^Variables are reported as mean (standard deviation). IKDC: International Knee Documentation Committee, ACLR: anterior cruciate ligament reconstruction, SA-ACLR: suture augmentation anterior cruciate ligament reconstruction

Concerning the post-operative complications, 4 (5.1%) patients in the standard ACLR group and 4 (4.4%) in the SA-ACLR group experienced graft retear requiring revision surgery within the first 24 months after the primary ACLR. The measured difference was not statistically significant between the two study groups (*P* > 0.05). No patient suffered from a contralateral ACL tear during the 24-month follow-up period. DVT was detected in 1 (1.1%) patient in the SA-ACLR group within 90 days after the operation and no patient in the standard ACLR group was complicated with DVT; however the difference in this complication did not achieve the statistical level of significance (*P* > 0.05). Surgical site infection requiring intervention occurred in 1 (1.3%) patient in the standard ACLR group within 90 days after the operation, who was successfully treated by intravenous antibiotics and arthroscopic joint lavage without a need for surgical debridement of the graft. Infection was not seen in any of the patients in the SA-ACLR group. This difference between groups regarding infection was not significant (*P* > 0.05). None of the patients developed chronic aseptic swelling of the knee in any of the groups during the follow-up period.

## Discussion

In this study we investigated the short-term PROMs and complications of patients with ACL injury operated with either standard ACLR or SA-ACLR. The main findings of this study were the significant improvement in all patients after ACLR, and significantly better PROMs in patients operated with SA-ACLR compared to the standard surgical method. However, regarding the ACLR complications, both approaches had similar results and adding the SA did not prevent more complications significantly.

Despite the ever-improving field of operative sports medicine, the trends in evidence suggest that the outcome of ACLRs with newly developed techniques might not be that much superior to the older methods, and evidence is inconclusive in many cases on choosing the optimal treatment [9]. This leads to an ongoing research on technical improvements that may translate to functional improvements in patients undergoing ACLR [9]. The origin of augmentation of a biologic graft goes back to the 1980s, and SA for ligament repair or reconstruction has recently been used in many anatomic sites including for ACLR, which most of them have had promising results [24–28]. Mackay et al. in 2015 first published the utilization of a synthetic suture tape (InternalBrace™) as an augmentation for primary ACL repair as well as other ligamentus components of knee, which was a newly introduced method as a suture tape compared to the older methods using augmentation devices for ACLR [29]. Although originally used to enhance the results of ACL repair, SA-ACLR theoretically aims to protect the vulnerable graft tissue during the healing and ligamentization process [30], and potentially may result in earlier rehabilitation and return to activity, and reduce the risk of graft retear [14].

The most notable finding of the present study is that the mean PROMs of IKDC and Tegner-Lysholm scores of patients treated using the SA-ACLR technique were significantly higher 24 months after the surgery compared to those treated using the standard ACLR. This finding means achieving better function, less pain, and fewer symptoms with this surgical method, which was comparable to similar evidence in the field. Bodendorfer et al. investigated the results of SA using InternalBrace™ (Arthrex, Naples, FL, USA) versus standard ACLR through a matched comparative analysis on a total number of 60 cases, and reported significantly improved PROMs, less pain, higher rates and earlier return to preinjury activity level with the SA compared to the results of standard ACLR using hamstring [15].

Likewise, Aujla et al. in their study of 66 augmented ACLRs with Ligament Augmentation and Reconstruction System compared to 130 standard ACLRs reported significantly higher Tegner scores and return to sports among the augmented ACLR patients one-year post-surgery, besides the mean of IKDC and Lysholm scores as 91.7 ± 8.5 and 93.8 ± 7.8, respectively in the augementation group, and 91.2 ± 9.4 and 94.0 ± 8.5 in the standard ACLR group; however, the difference between groups were not statistically significant (P-values 0.717 and 0.873, respectively), besides the lower retear rates during two years after surgery [31]. Ebert et al. prospectively followed 65 patients undergoing hamstring ACLR augmented with Ligament Augmentation and Reconstruction System for two years and reported the mean of IKDC and Lysholm scores as 91.6 ± 8.3 and 93.7 ± 8.1 respectively, showing statistically significant improvements in both scores comapared to pre-operative assessments (P-values 0.006 and 0.008, respectively) [32]. Of note, although all their patients were operated using the hamstring graft, unlike our study they harvested both the semitendinosus and gracilis grafts and used the Ligament Augmentation and Reconstruction System (Corin Group) construct as reinforcement [32].

In contrast to the promising results reported in literature, some studies found no positive impacts of augmentation on the results of ACLR. Drogset et al. followed 68 patients for 8 years and reported the mean Lysholm score as 84 in the augmentation group and 87 in the control group, and suggested no positive long-term benefits of using an augmentation device for ACLR [12]. Although the results of that study were not so much promising, it should be noted that heir technique was different from our study both in the graft used (bone-patellar tendon-bone) and the augmentation device (Kennedy, 3 M, St Paul, Minnesota), and their patients were operated between 1991 and 1993 [12]. So recent advances in arthroscopic surgical techniques might result in better results. Moreover, Peterson et al. in a clinical trial randomizing ACLR cases into two groups operated by bone-patellar tendon-bone as the graft for ACLR with and without a synthetic degradable augmentation device (polyurethane urea) and following them for 12 years, reported no significant difference in clinical outcomes of a synthetic augmentation device regarding the assessed Tegner score and the Knee injury Osteoarthritis Outcome Score (KOOS) between the two groups in non of the short-, intermediate-, and long-term follow up [13].

Biomechanically, whether the augmentation of the graft results in the enhanced strength has been controversial. In a recent biomechanical study on porcine knees, Lai et al. concluded that SA significantly increases the ultimate strength, yield strength, and cyclic displacement in a weakened graft model with 80% graft resection, but with no significant improvement in stiffness in intact ACL grafts [33]. The final recommendation of that study was the positive results of SA on ACLR in terms of graft integrity, that could be used to expand the future clinical application of this approach for ACLR [33]. On the other hand, Bachmaier et al. in a recnet biomechanical study on bovine ACL grafts demonstrated that SA significantly increases graft stiffness protecting the graft from excess load and reducing graft elongation leading to a lower risk of graft tear [34].

Our results showed graft retears rates of 5.1% in the standard ACLR and 4.4% in the SA-ACLR group requiring revision surgery 24 months postoperatively that was not a significant difference. We should consider that graft retear is a multifactorial phenomenon and the biomechanical properties of the graft are not responsible for this complication in all cases of retear [35]. Aujla et al. reported a 3.0% overall ipsilateral/contralateral graft retear rate for the augmented ACLR group and a 3.8% retear rate for the standard ACLR group during a 2-years follow up, and similar to our results, they failed to observe a statistically significant difference between the two methods [31]. Ebert et al. reported a 2.0% ACL retear rate at 7 months postoperatively and 3.0% contralateral ACL tear rate at the 12 months follow up [32]. We believe that in order to answer the question whether SA for ACLR results in lower clinical retear rates, we need clear surgical indications, a standardized technique, and a long-term prospective follow up.

Regarding the complications in the current study, one patient (1.1%) in the SA-ACLR group was diagnosed with DVT with no evidence of pulmonary embolism, while the observed between-group difference was insignificant. Forlenza et al. in a large-scale study on 11,977 patients undergoing standard ACLR reported the 30-day and 90-day venous thromboembolism incidence rates to be 1.0% and 1.2%, respectively [36]. It seems that the SA technique does not alter the risk of DVT after the operation. As mentioned earlier, all of our patients received 81 mg of ASA for 28 days as primary prophylaxis for DVT, although recent evidence questions the benefits of such approach and supports the use of primary pharmacologic prophylaxis only in high-risk patients undergoing orthopedic surgeries which should be adjusted by the condition of each case [37, 38].

Although adding a synthetic material to the graft might raise some solicitude regarding the infection rate, we had only one case of surgical site infection in a patient who was operated by the standard ACLR, and the infection was treated accordingly. None of the patients in the SA-ACLR group developed surgical site infection within the first 90 post-surgical days. None of our patients in both groups received oral postoperative prophylactic antibiotics. Ebert et al. reported one (1.5%) case of early superficial infection in their cohort of 65 augmented-ACLR patients [32]. Aujla et al. reported similar infection rates of about 1.5% in both augmented and standard ACLR groups with early superficial wound infection both treated with antibiotics [31]. Additionally, in a canine arthroscopic ACLR model study for synthetic augmentation of ACL graft, pathologic examination of the 10 augmented quadriceps tendons did not show any evidence of infection [39].

There may be two more concerns regarding synthetic SA of the ACL grafts. First is the stress shielding the biologic graft by the synthetic suture, and second is the risk of an inflammatory response to the intraarticular suture and subsequent chronic synovitis [13]. In a biomechanical full-construct model of ACLR, the investigators recommended that the suture-graft construct work in a load-sharing manner, and this arrangement grants persistent loading of the ACL graft, a process which is necessary for revascularization and remodeling of the susceptible graft [34]. To ensure the suture not to be tighter than the graft in this study, first we fixed the suture in full extension position of knee by manual tension using a metal screw as described before, and then fixed the graft in 30 degrees of the knee flexion. Finally, the ultimate suture tape and graft proportionate isometry were approved by the arthroscopic examination.

Other complications have been reported in studies on using augmentation devices for ACLR. In their 8-year follow-up results, Drogset et al. reported four (8.2%) patients in the augmented group and six (11.8%) in the control group to have swelling in the operated knee, although the observed difference was not significant [12]. We observed no such problem in any of our cases during the two-year follow-up period. We should note that the synthetic material used in their study was different [12], that could justify the variations in findings. The pathologic examination of the canine models in another study also showed no rejection of the synthetic intraarticular material [39]. In a biocompatibility study of intraarticular multistranded long-chain polyethylene suture tape in a preclinical canine ACL model, Smith et al. studied six FiberTape® grafts (Arthrex, Naples, FL), of which three were left intact and three were transected to mimic the “worst-case’’ scenario of frayed suture ends in clinical conditions, and their necropsy investigation at six months postoperatively revealed no gross pathology for periarticular tissues in any of the specimens of both intact and transected suture tapes [40]. They also reported no immune or foreign body reaction, or cartilage damage in any of the investigated canine models [40].

The introduction of suture augmentation in ACLR not only enhances patient outcomes but also presents a remarkable combination of cost and time efficiency. Unlike many advanced surgical methods that often come with substantial financial implications, suture augmentation incurs minimal expenses. By utilizing a nonabsorbable suture, a widely available and cost-effective material, this technique significantly reduces financial strain in healthcare settings, especially in low- and middle-income countries like the origin of this study. This economic feasibility is crucial in the current healthcare landscape, emphasizing the importance of optimizing resources and minimizing unnecessary expenses. Moreover, the procedure seamlessly integrates into the existing surgical workflow, emphasizing the collaboration between team members. While the orthopedic surgeon focuses on the intricate aspects of the ACLR procedure, the surgeon’s assistant prepares the suture material concurrently, ensuring no significant additional time is required. This synchronized effort minimizes concerns about prolonged surgical time, maintaining the overall procedure duration at an optimal level.

Crucially, our study demonstrates that this innovative approach not only enhances short-term functional PROMs but does so without imposing substantial financial burdens. Emphasizing its simplicity and collaborative nature, the technique proves to be both cost-effective and time-efficient. Our study does not intend to endorse a specific product or procedure; instead, it aims to offer valuable scientific insights. By presenting this alternative technique, which combines simplicity with cost-effectiveness, we contribute to improving patient outcomes.

This study had some limitations. The first and most important one was the retrospective design of study that limits the level of evidence and may put the results at risk of some bias, besides the fact that due to temporal differences between two study cohort, the comparison of their outcomes might be influenced by some external factors. Second, although the demographic and clinical characteristics of the patients in the two groups were insignificantly different, they were not randomly allocated to the intervention groups. Third, the duration of follow-up in our study was limited to two years and it is unknown whether the observed trends in PROMs will still exist in the long run. Fourth, although we prescribed the same rehabilitation protocol for all of our patients, they were treated in different physiotherapy clinics by different therapists, and the patients’ compliance and adherence to the program are somewhat unclear to us. Fifth, the patients of these two groups were operated in two different time periods and a temporal association might be seen with the overall improved technique and instruments of arthroscopic surgery. Sixth, the size of graft diameter was made after careful consideration of both the available evidence and the practices in our institution, which might be different in other centers and may affect the surgical outcomes. In spite of all these limitations, the current study is among the most updated evidence on the use of SA in ACLR in a country with limited healthcare resources, and the findings would be beneficical to improve the outcomes of patients undergoing ACLR.

## Conclusion

The present study demonstrated that using SA for ACLR improves short-term functional outcomes and PROMs, and does not increase the risk of complications including infection, chronic synovitis, and DVT. Besides, this approach does not decrease the risk of graft retear compared to the standard hamstring ACLR. Better PROMs associated with the SA-ACLR technique offers this approach as a superior and viable option to treat patients with ACL injuries.

## Data Availability

The datasets analyzed in the current study are available from the corresponding author upon reasonable request.

## Abbreviations

ACLR: Anterior cruciate ligament reconstruction
PROMs: Patient-reported outcome measures
SA: Suture augmentation
IKDC: International Knee Documentation Committee
ACL: Anterior cruciate ligament
UHMWPE: Ultra-high molecular weight polyethylene
ROM: Range of motion
ASA: Acetylsalicylic acid
DVT: Deep vein thrombosis
BMI: Body mass index
SD: Standard deviations
ANOVA: Analysis of variance
KOOS: Knee injury Osteoarthritis Outcome Score
